# High-Throughput Sequencing Assists Studies in Genomic Variability and Epidemiology of Little Cherry Virus 1 and 2 infecting *Prunus* spp. in Belgium

**DOI:** 10.3390/v11070592

**Published:** 2019-06-29

**Authors:** Rachid Tahzima, Yoika Foucart, Gertie Peusens, Tim Beliën, Sébastien Massart, Kris De Jonghe

**Affiliations:** 1 Plant Sciences, Fisheries and Food (ILVO), Flanders Research Institute for Agriculture, 9820 Merelbeke, Belgium; 2 Department of Integrated and Urban Phytopathology, University of Liège (ULg) - Gembloux Agro-Bio tech, 5030 Gembloux, Belgium; 3 Department of Zoology, Proefcentrum Fruitteelt (pcfruit), 3800 Sint-Truiden, Belgium

**Keywords:** *Closteroviridae*, *Ampelovirus*, *Velarivirus*, genetic variability, HTS, total RNA, fruit trees and plant viruses, molecular evolution, RNA virus phylogeny

## Abstract

Little cherry disease, caused by little cherry virus 1 (LChV-1) and little cherry virus 2 (LChV-2), which are both members of the family *Closteroviridae*, severely affects sweet (*Prunus avium* L.) and sour cherry (*P. cerasus* L.) orchards lifelong production worldwide. An intensive survey was conducted across different geographic regions of Belgium to study the disease presence on these perennial woody plants and related species. Symptomatic as well as non-symptomatic *Prunus* spp. trees tested positive via RT-PCR for LChV-1 and -2 in single or mixed infections, with a slightly higher incidence for LChV-1. Both viruses were widespread and highly prevalent in nearly all *Prunus* production areas as well as in private gardens and urban lane trees. The genetic diversity of Belgian LChV-1 and -2 isolates was assessed by Sanger sequencing of partial genomic regions. A total RNA High-Throughput Sequencing (HTS) approach confirmed the presence of both viruses, and revealed the occurrence of other *Prunus*-associated viruses, namely cherry virus A (CVA), prune dwarf virus (PDV) and prunus virus F (PrVF). The phylogenetic inference from full-length genomes revealed well-defined evolutionary phylogroups with high genetic variability and diversity for LChV-1 and LChV-2 Belgian isolates, yet with little or no correlation with planting area or cultivated varieties. The global diversity and the prevalence in horticultural areas of LChV-1 and -2 variants, in association with other recently described fruit tree viruses, are of particular concern. Future epidemiological implications as well as new investigation avenues are exhaustively discussed.

## 1. Introduction

Little cherry disease (LChD) is an economically important viral disease causing a wide range of phenotypic responses and significant reduction of fruit yield and quality in sweet (*Prunus avium* L., family Rosaceae) and sour cherry (*Prunus cerasus* L.) field stock nurseries and commercial orchards. Two distinct (+)ssRNA viruses, found in single or mixed infections, are the associated causal agents of LChD. The species Little cherry virus 1 (LChV-1) is a member of the recently described genus *Velarivirus* within the family *Closteroviridae* [1,2], while the species Little cherry virus 2 (LChV-2) belongs to the genus *Ampelovirus* in the same family [3,4].

Sweet cherry is an economically important stone fruit species in Belgium and Europe and is exploited not only for its fruits, but also for propagation material and rootstocks in stock nurseries [5,6,7]. Several *Prunus* spp., including the ornamental flowering cherry (*P. serrulata* Lindl.), can be infected, yet often latently, with different plant viruses including both LChV-1 and LChV-2 viruses. In sweet cherry, LChD is expressed as a wide array of phenotypic features ranging from asymptomatic to showing severe leaf and fruit symptoms such as premature reddening or bronzing, as well as the development of small fruits, uneven ripening and an insipid taste. Symptoms are highly variable among cherry cultivars and are strongly influenced by season, weather and growing conditions ([8] and references therein), leading to significant yield losses and poor-quality fruit. This variability limits the ability of growers to specifically associate poor fruit development with LChD.

Apart from vegetative propagation of systemically infected plant materials in stock nurseries, the most common mechanism of LChV-1 and -2 transmission is mechanical, by direct grafting of infected budwood onto scion or rootstocks. No insect vector has been identified so far for LChV-1, in contrast to LChV-2 for which at least two distinct species of mealybugs (Hemiptera, *Pseudococcidae*) are known to naturally transmit the virus inefficiently in a semi-persistent mode and infrequent way, namely the grape mealybug (*Pseudococcus maritimus* Ehrhorn) and the apple mealybug (*Phenacoccus aceris* Signoret). Neither LChV-1 or LChV-2 are pollen- or seed-transmitted [2,3,4,8,9]. LChD was first reported in the USA and Canada in the late 1930s, occasionally in association with the Western X phytoplasma disease [8], and is now present on a broad range of stone fruit species in many other cherry-growing areas of the world [2,10,11,12,13,14,15,16,17,18,19,20,21,22,23,24], including Belgium [25,26].

The wide intra- and inter-host genetic diversity of LChV-1 and LChV-2 has been characterized only in a few countries [2,3,4,27,28,29,30,31,32,33,34,35,36]. Both viruses are confirmed to be present in Belgium, however no comprehensive phylogenetic characterization of the LChD has been published so far. The molecular diversity and evolutionary relationships of the Belgian LChV-1 and -2 isolates were assessed and characterized in their natural host plants based on comparative partial and full genome sequencing. This study was supported by total RNA High-Throughput Sequencing and provides useful information on the prevalence and genetic variability of both LChV-1 and LChV-2, including the presence of other *Prunus*-infecting viruses in Belgium, which enhances our understanding of little cherry disease.

## 2. Materials and Methods

### 2.1. Plant Material Collection and Samples Preparation

Field surveys were conducted from spring 2014 to summer 2017 in four different northern Belgian (Flanders) regions (West Flanders, East Flanders, Flemish Brabant and Limburg, see map in Supplementary Material S1). The incidence of LChV-1 and LChV-2 was investigated in both Belgian cherry and plum orchards and in flowering cherry trees. Leaf and branch samples were mainly collected in orchards with 1%–30% symptomatic cherry trees showing LChD-like reddening on their leaves, as well as from symptomless trees and in seemingly healthy orchards. From each tree, a pool of leaves, stems and bark tissue from annual shoots and roots was collected and prepared as a composite sample. Total RNA was isolated from cambial scrapings of the leaf midrib and from branch samples using Spectrum™ Total Plant RNA Kit (Sigma-Aldrich, Overijse, Belgium) according to the manufacturer’s instructions. The purified total RNA quality and quantity were assessed using a Nanodrop ND-1000 spectrophotometer (ThermoFisher Scientific, Waltham, MA, USA). Additionally, total RNA from virus-free (virus tested) greenhouse plants was used as the non-infected control. The final concentration of total RNA was adjusted using a Nanodrop to 50 ng µL^−1^ with RNase-free water and subsequently diluted tenfold in nuclease-free Milli-Q^®^ water (Merck Millipore, Burlington, MA, USA). RNA extracts were stored at −70 °C.

### 2.2. RT-PCR and Sequencing

Prior to RT-PCR, cDNA was synthesized from a representative selection of RNA samples with an iScript cDNA synthesis kit (Bio-Rad, Hercules, CA, USA) following the supplier’s instructions. The generated cDNA was used as a template for different PCR reactions using specific primers [22,23,29,30] or primers designed for this study (see Table S1). Amplifications were carried out in a total volume of 25 μL of PCR mixture containing FastStart^™^ Taq DNA Polymerase reaction mix (Roche, Mannheim, Germany) in an ABI9700 GeneAmp Thermal Cycler (Applied Biosystems, Foster City, CA, USA). Target-specific amplifications were visualized by high-resolution capillary electrophoresis with a Qiaxcel Advanced system (Qiagen, Antwerp, Belgium) according to the manufacturer’s instructions or by agarose gel electrophoresis. PCR products were gel-purified with a Nucleospin PCR Clean-up kit (Macherey–Nagel, Düren, Germany) and were bidirectionally sequenced (Macrogen Inc., Amsterdam, The Netherlands). RT-PCR detection of cherry virus A, prunus virus F and prune dwarf virus was carried out following published protocols [10,35,37].

### 2.3. Sequences and Phylogenetic Analysis

The obtained Sanger sequences from the RdRp and CP genomic regions were assembled, analyzed and aligned using the BioNumerics 7 (Applied Math version 7.6.1). Sequence similarity was confirmed using the BLASTn program in GenBank (https://blast.ncbi.nlm.nih.gov/Blast.cgi). In addition to Belgian sequences, a selection of nt sequences of the RdRp and CP genes of all representative LChV-1 and -2 from different countries and host plants was retrieved from GenBank, aligned and used for phylogenetic analyses and molecular evolutionary genetics analysis with the MEGA software package version 7.0 [38,39] (Table S2). The deduced amino acid sequences of the RdRp and coat protein gene were obtained with the open reading frame finder ORFfinder online tool from NCBI.nih.gov/ORFfinder. Phylogenetic trees were generated from nucleotides alignments of partial and full genome sequences using maximum likelihood (ML) algorithms with assessment of the confidence of branching patterns by bootstrap analysis with 1000 pseudo-random iterations to test the robustness of the internal branches. The identification and accession numbers of the LChV-1 and LChV-2 isolates of this study, together with all other *Prunus* related viruses that were included in this analysis, are available in the GenBank database under taxid: 217686 and taxid: 154339 (Tables S2 and S3).

### 2.4. Total RNA Sequencing and Bioinformatics Analysis

Total RNA was extracted from 100 mg of fresh leaf material using the Spectrum Total Plant RNA Kit (Sigma Aldrich N.V). Quantification and quality controls were done with Nanodrop ND-1000 spectrophotometer and Quantus (QuantiFluor^®^ RNA System kit (Promega Benelux B.V.) followed by RNA-purification (NucleoSpin^®^ RNA Clean-up XS; Machery-Nagel, Germany). Library preparation and rRNA-depletion were done externally (Admera Health, South Plainfield, NJ, USA) using the NEBNext^®^ Ultra™ RNA Library Prep Kit for Illumina^®^ and Ribozero Plant kit, respectively, followed by NextSeq sequencing (2 × 150 bp read length, 2 × 20 M reads per sample). The obtained sequence reads were subjected to quality filtering, adapter removal and a standardized bioinformatics analysis strategy using Cutadapt, Pear, SortmeRNA and the VirusDetect pipeline [40]. The consensus sequences of the complete genomes were obtained through reference mapping in CLC Genomics Workbench 12 (Qiagen, Vedbæk, Denmark).

## 3. Results

### 3.1. Incidence and Etiology of LChV-1 and LChV-2 in Prunus Trees Grown in Belgium

In total, 88 (29.3%) and 39 (13.0%) out of 300 samples tested positive by RT-PCR for LChV-1 and LChV-2, respectively, and were sequenced. In all surveyed Belgian locations (Figure S1, Table S4), LChV-1 and -2 infections were found more frequently in sweet cherry trees than in ornamental trees. LChV-1 was also detected in one plum cultivar. In infected cherry trees, leaf symptoms like premature leaf reddening and interveinal bronzing of the upper leaf surface were observed with variation in symptom expression between cultivars. No symptoms were associated with plum and ornamental cherry trees.

### 3.2. Diversity and Comparison of LChV-1 and LChV-2 Sequences Encoding RdRp and CP Gene

In total, 155 sequences from LChV-1 and -2 isolates (116 sequences from our study and 39 from GenBank) were retrieved. The LChV-1 RdRp domain encoding region of 72 Belgian isolates and the LChV-1 CP gene of 16 isolates were partially sequenced, exhibiting limited sequence diversity (with 98%–99% similarity). The results also clearly showed a higher variability of LChV-1 based on the CP gene (84%–99% similarity) when compared with the RdRp gene, showing more variability towards the 3’ end. In some samples, mixed LChV-1 infections occurred with CVA. The LChV-2 RdRp and CP gene were Sanger sequenced and compared with all LChV-2 sequences available in GenBank. The LChV-2 RdRp nt sequences of these isolates shared 100% and 99% identity with each other, 97%–99% with LC5 (Accession No. AF416335, Canada) and 95% with M75 (KT369316, Croatia) and 98% with USA6b (AF531505, USA), respectively. The CP sequence of the Belgian LChV-2 isolates (454 bp) shared 94%–98% identity with each other, 98% with LC5 and C14 (EU153101, Poland) and 100% with USA6b. 

### 3.3. Phylogenetic Inference Reveals the Diversity of LChV-1 and LChV-2 in Belgium

**Figure 1:**
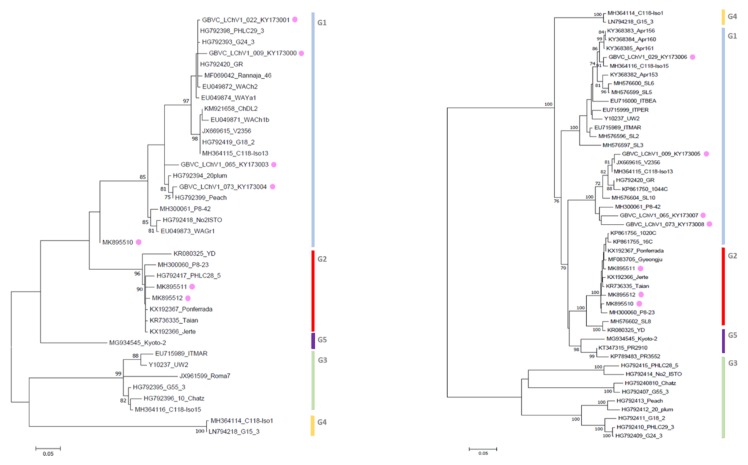
Maximum likelihood phylogenetic tree inferred from partial RdRp (left) and CP (right) nucleotide sequences of the LChV-1 Belgian (purple dot) and other LChV-1 isolates. The GenBank accession numbers of the sequences are indicated together with the isolate name, host plant and cultivar. MEGA 7.0 analysis included most of the available LChV-1 sequences. The phylogenetic clusters are delineated with vertical bars. Branch lengths on the phylogenetic tree represent the genetic distance the numbers at the branches represent the percentage of replicates in which the topology of the branch was observed after 1000 bootstrap replicates.

**Figure 2:**
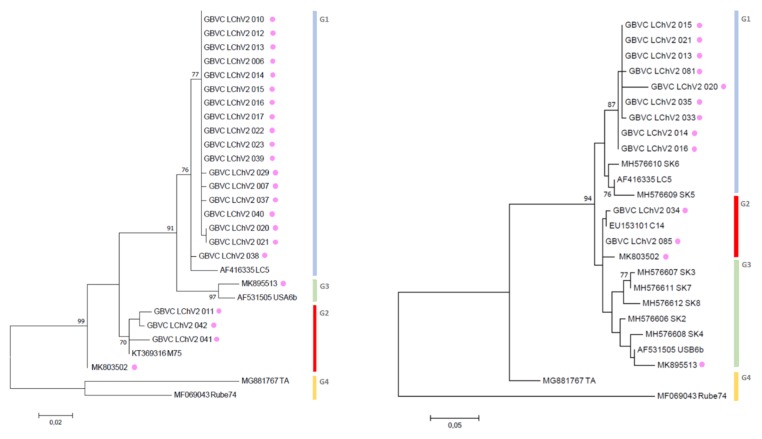
Maximum likelihood phylogenetic tree inferred from partial RdRp (left) and CP (right) nucleotide sequences of the LChV-2 Belgian (purple dot) and other LChV-2 isolates. The GenBank accession numbers of the sequences are indicated together with the isolate name, host plant and cultivar. MEGA 7.0 analysis included most of the available LChV-2 sequences. The phylogenetic clusters are delineated with vertical bars. Branch lengths on the phylogenetic tree represent the genetic distance the numbers at the branches represent the percentage of replicates in which the topology of the branch was observed after 1000 bootstrap replicates.

For LChV1, maximum likelihood phylogenetic trees derived from the aligned partial RdRp, CP and complete genome nucleotide sequences from our study and from NCBI provided key information about their relationship within the LChV-1 group. Firstly, according to RdRp and CP nt sequences and using the same readily published phylogenetic methodology [2], we could reconstruct the entire LChV-1 phylogenetic landscape with the nt sequence of all LChV-1 Belgian isolates grouped into two distant large sub-clusters, closely related (97%–100% nt identity) to other GenBank sequences of LChV-1 from Greece (No2ISTO, HG792418) and Germany (V2356, JX669615) belonging to Group 1 and 2 (Figure 1 and Figure S2), in accordance with earlier studies [2,36,41]. Secondly, given the extensive spread of these notably divergent LChV-1 isolates infecting different *Prunus* species across Belgium and the lack of congruence according to the geographic or host-plant origin, these observations suggest that the virus was not recently introduced to Belgium. For LChV-2, similarly, maximum likelihood phylogenetic trees based on RdRp and CP partial nt sequence also identified different clusters divided into different sub-groups. This clustering into subgroup was confirmed by the partial CP and full genome nt sequence phylogenetic analysis as well (Figure 2 and Figure S2). Belgian LChV-2 sequences were highly homogenous and genetically closely related (97%–99% nt identity) to representative isolates LC5 (AF416335), C14 (EU153101), USA6b (AF531505) and M75 (KT369316). 

### 3.4. Total RNA HTS also Revealed the Presence of CVA, Prune dwarf viruses (PDV) and PrVF in Belgium

**Figure 3:**
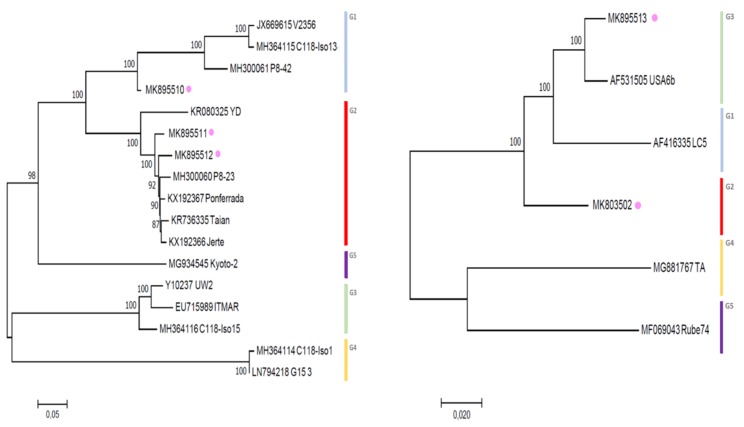
Maximum likelihood phylogenetic trees inferred from the full genome nucleotide sequences of LChV-1 (left) and LChV-2 (right) isolates. All isolates from Genbank are reported with their accession numbers followed by their names. Belgian isolates are indicated with a purple dot. The numbers above or below each branch are the nonparametric bootstrap (NPB) values given as percentages of 1000 replicates.

High-throughput sequencing was used to retrieve the full genomes of diverse Belgian LChV-1 (Accession No. MK895510-12) and LChV-2 (Accession No. MK803502, MK895513) representative isolates. In addition, their entire nt genomes (Figure 3), together with the genomes of three other co-infecting *Prunus* spp. viruses, namely CVA (*Capillovirus*, *Betaflexiviridae;* Accession No. MK847263-65), PDV (*Ilarvirus*, *Bromoviridae;* Accession No. MK834274-76) and the recently discovered PrVF (*Fabavirus*, *Secoviridae;* Accession No. MK834285-87), were identified and analyzed (Figures S2–S5). The presence of CVA, PDV and PrVF was also confirmed by conventional RT-PCR with specific primers and validated by Sanger sequencing (Tables S1–S3, and ref. [10,42,43,44].

## 4. Discussion and Conclusions

Many *Prunus* species are known to host a wide spectrum of viral populations and to frequently harbor mixed infections [8,43]. In this study, we demonstrated that the little cherry disease, caused by LChV-1 and LChV-2 was widely distributed in many popular *Prunus* varieties, including ornamental *Prunus* spp., and is present in all major cherry growing areas in Belgium. This is the first comprehensive epidemiological investigation on the prevalence, distribution and molecular diversity of both LChV-1 and -2 isolates from stone fruit trees in Belgium. LChV-1 and -2 infections were frequent in sweet cherry trees and most often associated with described symptoms [8]. This study is also the first report of the asymptomatic presence of LChV-1 infections in ornamental cherry species (*P. serrulata* cv. Amanogowa and *P. serrulata* cv. Lannesiana) [45] and symptomatic sour cherry trees (*P. cerasus*) grown in Belgium. The LChV-1 and -2 incidence and observed variability in all surveyed regions can mainly be attributed to the contribution of different plant material sources, their co-infectious status and their mode of propagation in long-established orchards. In a context of rapid and widespread disease worldwide, aspects like multiple infections in symptomless plant reservoirs are readily known to play a key epidemiological role [8,21,27,28,29,30,31,32,33,34,35]. Severe damage has occurred on sweet and sour cherry trees on which the LChD was observed, often leading, together with possible other biotic and abiotic factors, to full tree decline in devastated old orchards, as has been reported for most sensitive sweet cherry cultivars ([8], Supplementary Material). All other sampled *Prunus* species were confirmed to be latently infected. Their etiology varied considerably between scion and rootstock varieties and also according to season, year of sampling and sometimes co-infection as observed in earlier reports [2,3,8,21,22,27,28,29,30,31,32,33,34,35,36,41].

The phylogenetic analyses confirmed our hypothesis that the LChV-1 isolates are unlikely to have been derived from the same ancestor. They more likely resulted from multiple introductions over time. Our evolutionary phylogenetic inferences have reproduced similar results to recently published phylogenetic analyses [2,27,28,36,41], therefore supporting the detailed molecular characterization of this study. Additionally, no correlation could be found between the geographic locations, host species or cultivars, etiology, and the clades position of the LChV-1 and LChV-2 sub-clusters in the phylogenetic trees, which also confirmed earlier reports [2,41,46,47,48,49]. Taken together, our study of the genetic diversity of Little cherry virus in Belgium demonstrated that LChV-1 and -2 isolates formed distinct but congruent evolutionary groups with a clear yet limited molecular evolution and diversity.

In Belgium, the presumably natural vector-borne spread of LChV-2 was reported in the early 1980s, suggesting selective pressure and strong virus adaptation to the local described coccoid vectors [25], probably explaining the lower variability among the Belgian LChV-2 isolates and likely supported by extensive global exchange of infected propagation material [29,30,31,32,33]. In Europe and North America, only few reports on LChV-2 vector transmission have been published, namely with *Macrosteles fascifrons*, *Pseudococcus maritimus* and *Phenacoccus aceris* [8,9,25,50]. Furthermore, in absence of a known vector for LChV-1, the massive production, trade and sharing of pathogen-tested propagation material from Japan, on one hand, and, the import of susceptible cherry cultivars from North America and Canada, on the other hand, most likely served as the major viral disease introduction pathway. Host and potential vectors may be found to be important evolutionary and epidemiological factors shaping the genetic variability of these diverse viral populations when considered as collection of variants in the context of viral quasispecies. This would emphasize the multiple facets of this disease complex in accordance with the general trend observed among viruses members of the family *Closteroviridae* [46,47,48,51,52,53,54,55,56,57]. Despite the general high potential for variability of most ss(+)RNA viruses, most members of the family *Closteroviridae*, like most RNA virus populations analyzed so far, are genetically stable with relatively moderate intra-taxonomical diversity [55,56]. The observed genetic diversity within the Belgian LChV-1 isolates was lower than for most LChV-1 isolates reported so far and was similar to another *Velarivirus,* namely GLRaV-7 [56,58]. Germplasm influxes were common in Belgium but are not yet regulated by a national certification scheme, which makes causal association between the occurrence of LChD and the origin of specific cultivars difficult to infer. This is consistent with other studies on the genetic variability of LChV-1 and can enlighten us on the current phytosanitary situation [2,27,28,29,30,31,32,33,34]. In Belgium, no specific phytosanitary measures were implemented in order to limit LChV-1 and LChV-2 introduction and spread. Nevertheless, the spread of LChV-1 and LChV-2 -infected propagation and planting material across Europe, together with the presence of (potential) vectors, will challenge the ability of horticultural systems to quickly shift towards resistant cultivars and will force an increase towards efficiency of phytosanitary measures through enforced *Prunus* certification programs [37,49,59,60].

Globally, an increased number of LChV-1 and LChV-2 isolate sequences are deciphered using HTS, thus it will be possible to develop improved diagnostic tools for reliable detection as well as to sequence additional whole genomes of LChV-1 and LChV-2 variants from a wide host range. Moreover, through extensive HTS investigations, it will become possible to identify new genotypes, to generate detailed biological information about these viral variants communities and to unveil non-described viral species and as such generate new insights on the epidemiological and etiological implications of the little cherry disease for the *Prunus* horticultural industry stakeholders and regulators [37,49,59,60,61,62]. In this study, total RNA HTS helped to detect, identify and characterize both LChV-1 and LChV-2 viruses in several representative samples. Additionally, it assisted to construct new genomes, at the same time revealing the presence of mixed infections with CVA (mechanically and seed transmitted), PrVF (probably aphid transmitted [42]) and PDV (no known vector and seed transmitted), which might also play a non-trivial role in symptomatology and epidemiology, while further hampering the virus-symptoms correlations in these woody perennial crops [42,43,44,50,63,64]. Also, regarding the use of HTS analysis in epidemiological studies, it is important to quantify the read counts of specific viruses in each sample. Using recent open source pipelines [65], the normalized read counts may reflect the relative abundance of specific viral RNAs within samples and provide valuable information such as the relative stability of viral RNA fragments within the whole genome and the within-population variations of the same virus species. In addition, the read counts could reveal the prevalence of different *Prunus*-associated viruses (LChV-1, LChV-2, CVA, PDV, PrVF, etc.) within a single sample and help elucidate several evolutionary and epidemiological questions. Future extensive biological indexing performed with a larger number of LChV-1 and LChV-2 isolates on different host species and which takes intra-host spatio-temporal population variations and these co-infections aspects into account will surely help to better clarify the complexity of symptom development. Their exact respective and cumulative role in etiology and synergism (or antagonism) in disease development, together with their epidemiology requires further urgent investigation. The combination of known and unknown (a) biotic factors and viral-host intrinsic features may considerably impact viral communities. The study of these factors (e.g., mutation rates, recombination events, SNPs, RNAs abundance, environment, cultivars, season) through comprehensive bioinformatics analysis is important for the design of sound disease management within plant health research.

Our findings provide a useful foundation for evaluating the epidemiological characteristics of LChD in Belgium and decisive information for nursery certification programs as well as for the design of sustainable integrated pest management strategies. Finally, identifying LChV-1 and LChV-2 variants through their whole genomic sequence recovery may facilitate the understanding of the *Prunus* virome interaction, dynamics and underline the distinctive phenotypic features of viruses associated with this disease. The occurrence of CVA and PDV in *Prunus* orchards was generally known, yet PrVF was detected and described for the first time in Belgium. Further extensive HTS investigative work is currently being undertaken to unravel the global intra- and inter-host diversity of the *Prunus* virosphere in light of LChD.

## Supplementary Materials

The following are available online at https://www.mdpi.com/1999-4915/11/7/592/s1
