# Supplementary material for: High-Throughput Sequencing Assists Studies in Genomic Variability and Epidemiology of Little Cherry Virus 1 and 2 infecting Prunus spp. in Belgium
Source: Viruses. 2019 Jun 29;11(7):592. doi: 10.3390/v11070592 (PMC6669712; doi:10.3390/v11070592)
Supplement: Supplementary file 1 [file viruses-11-00592-s001.zip › Tahzimaetal2019 Viruses SupplMat FiguresMapPhylogeneticData 140619 RT 518682.docx]

*
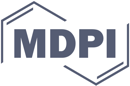
*
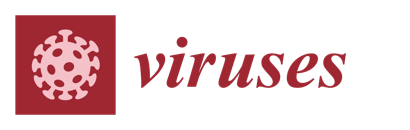
***Supplementary Material Figures***

**HTS assists Genomic Variability and Epidemiology Studies of Little Cherry Virus 1 and 2 infecting *Prunus* spp. in Belgium**

**Rachid Tahzima ^1,3^, Yoika Foucart ^1^, Gertie Peusens ^2^, Tim Beliën ^2^, Sébastien Massart ^3^, and Kris De Jonghe ^1,*^**

^1^ Flanders Research Institute for Agriculture, Fisheries and Food (ILVO), Plant Sciences, 9820 Merelbeke, Belgium. [rachid.tahzima@ilvo.vlaanderen.be](mailto:rachid.tahzima@ilvo.vlaanderen.be), [rachid.tahzima@uliège.be](mailto:rachid.tahzima@uliège.be), yoika.foucart@ilvo.vlaanderen.be

^2^ Proefcentrum Fruitteelt (pcfruit), Department of Zoology, 3800 Sint-Truiden, Belgium. [tim.belien@pcfruit.be](mailto:tim.belien@pcfruit.be), [gertie.peusens@pcfruit.be](mailto:gertie.peusens@pcfruit.be)

^3^ University of Liège (ULg) - Gembloux Agro-Bio tech, Department of Integrated and Urban Phytopathology, 5030 Gembloux, Belgium. [sebastien.massart@uliège.be](mailto:sebastien.massart@uliège.be)

*Corresponding author: K. De Jonghe; E-mail: [kris.dejonghe@ilvo.vlaanderen.be](mailto:kris.dejonghe@ilvo.vlaanderen.be)


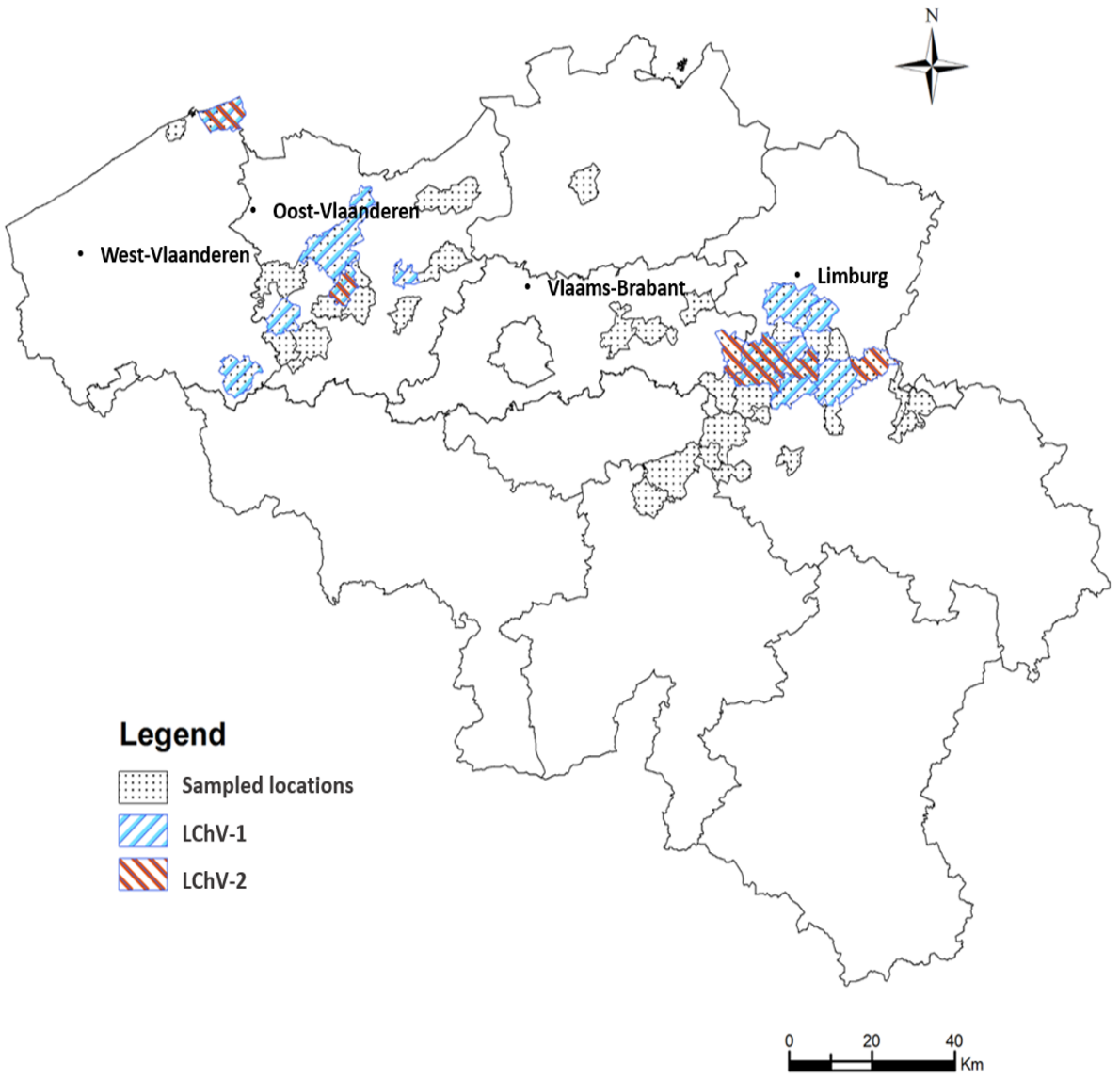


**Figure S1**. Distribution map of Belgium showing LChV-1 and LChV-2 collection sites and regions of occurrence for this epidemiological survey. Map was constructed using the ArcGIS 10.0 software (ESRI® ArcMAP™ 2010).


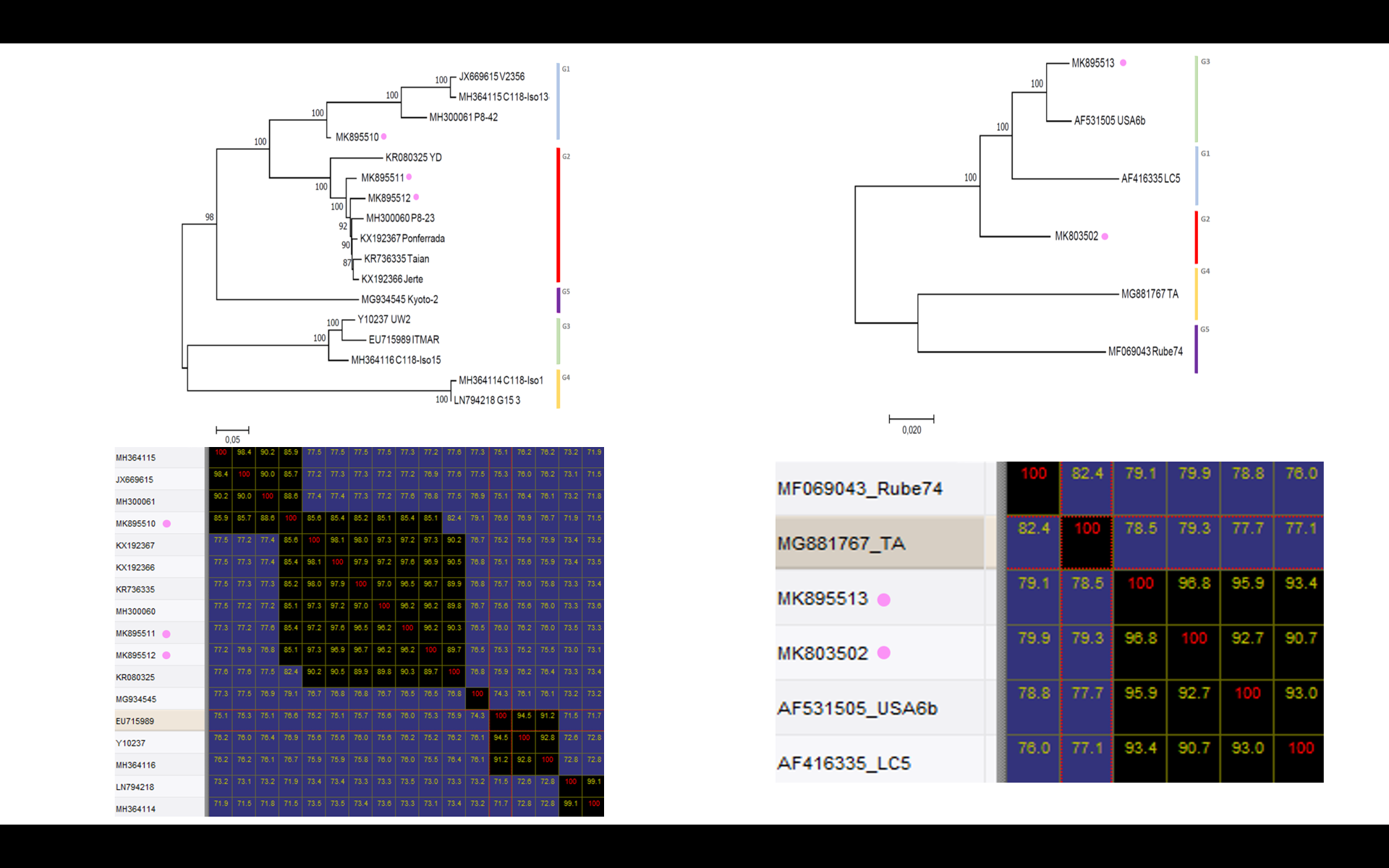


**Figure S2.** (A) Maximum likelihood phylogenetic trees inferred from the full genome nucleotide sequences of LChV-1 (left) and LChV-2 (right). Belgian isolates are indicated with purple dot (GBVC). All isolates from Genbank are reported with their accession numbers followed by their names. The numbers above or below each branch are the nonparametric bootstrap (NPB) values given as percentages of 1000 replicates. (B) Percent identity matrix from pairwise comparison of full genome nucleotide sequences available at GenBank for LChV-1 and LChV-2 , respectively.


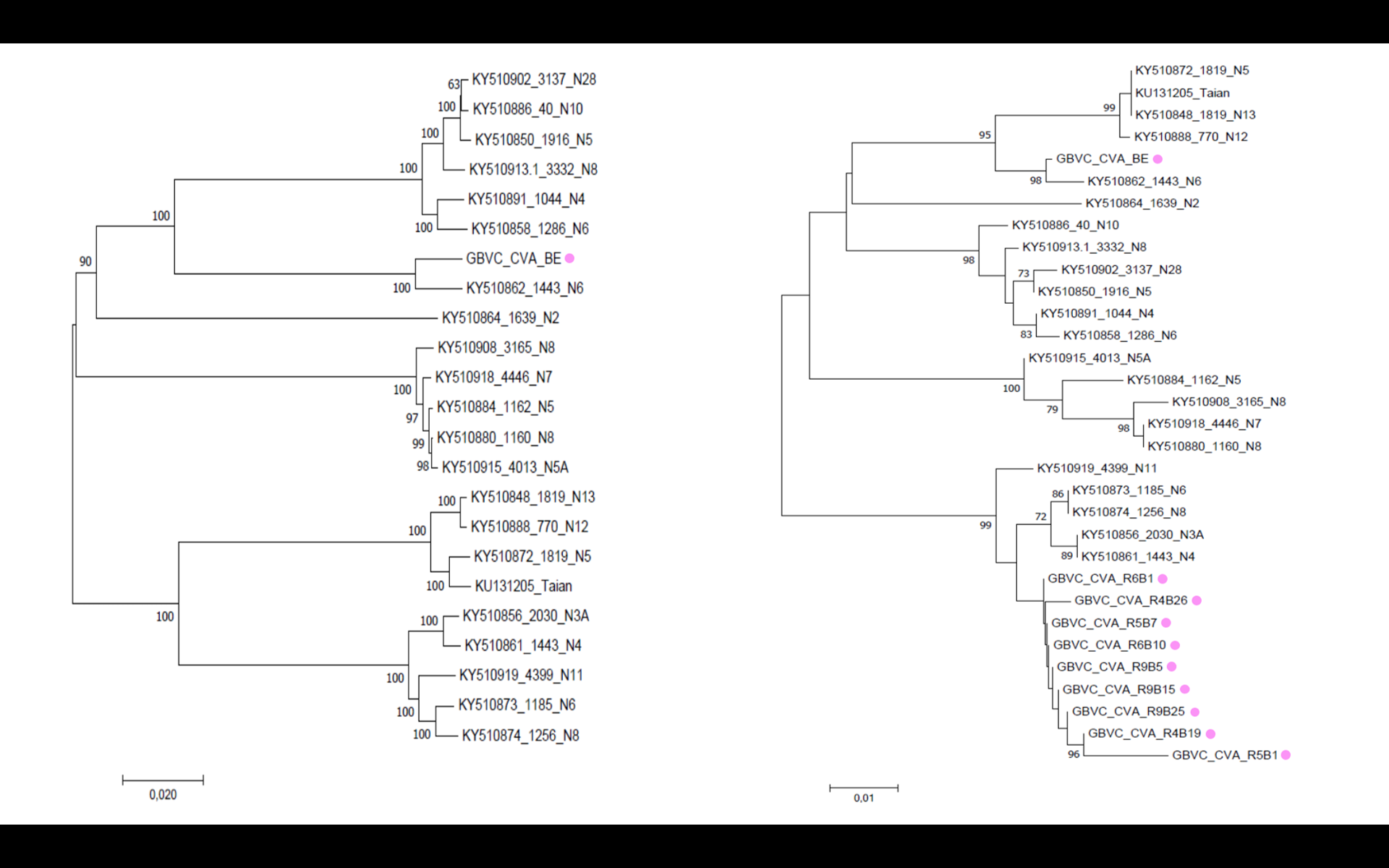

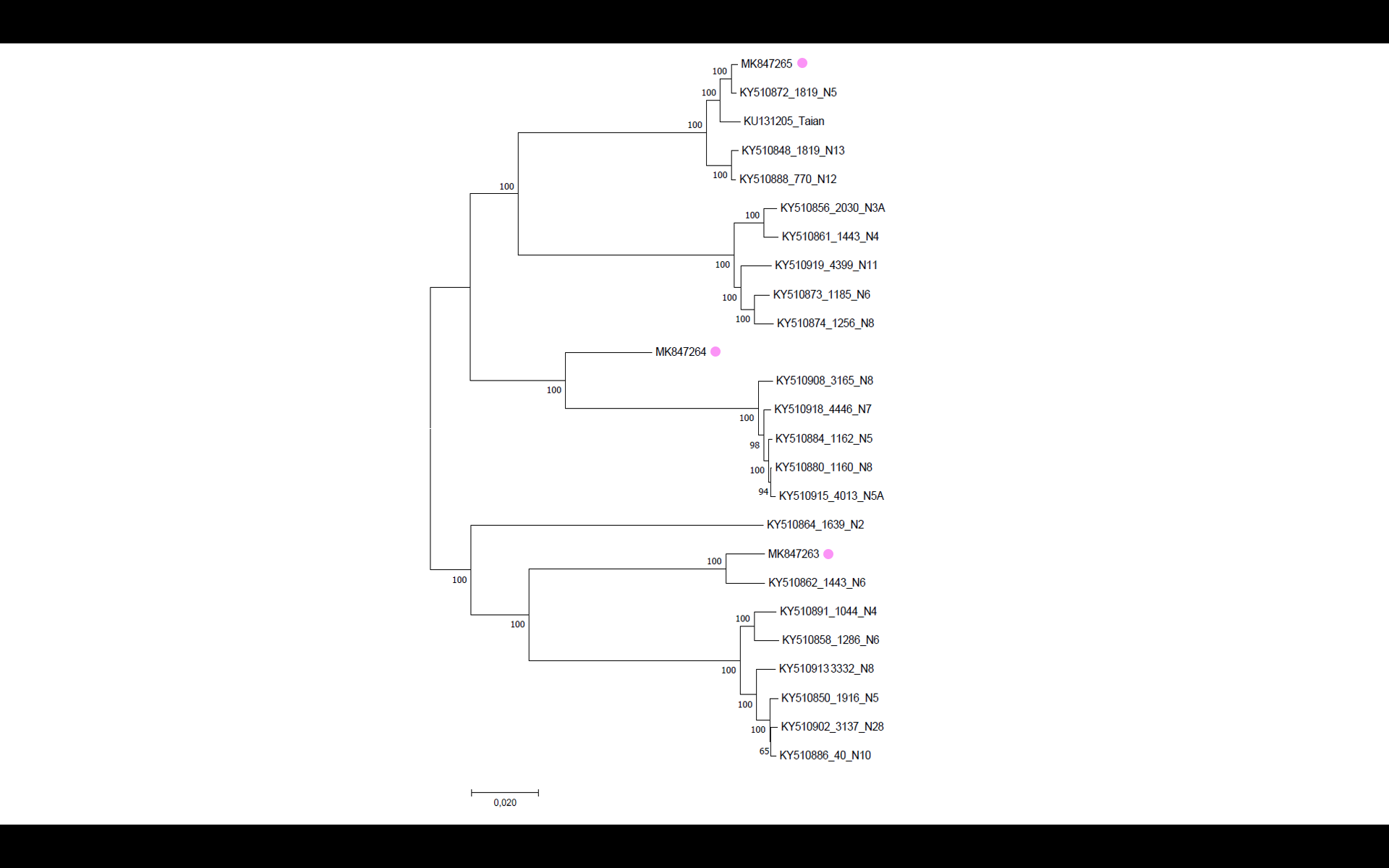


**Figure S3.** Maximum likelihood phylogenetic trees inferred from the full genome (left) and partial (right) nucleotide sequences of CVA isolates (Belgian isolates are indicated with a purple dot). All isolates from Genbank are reported with their accession numbers followed by their names. The numbers above or below each branch are the nonparametric bootstrap (NPB) values given as percentages of 1000 replicates.


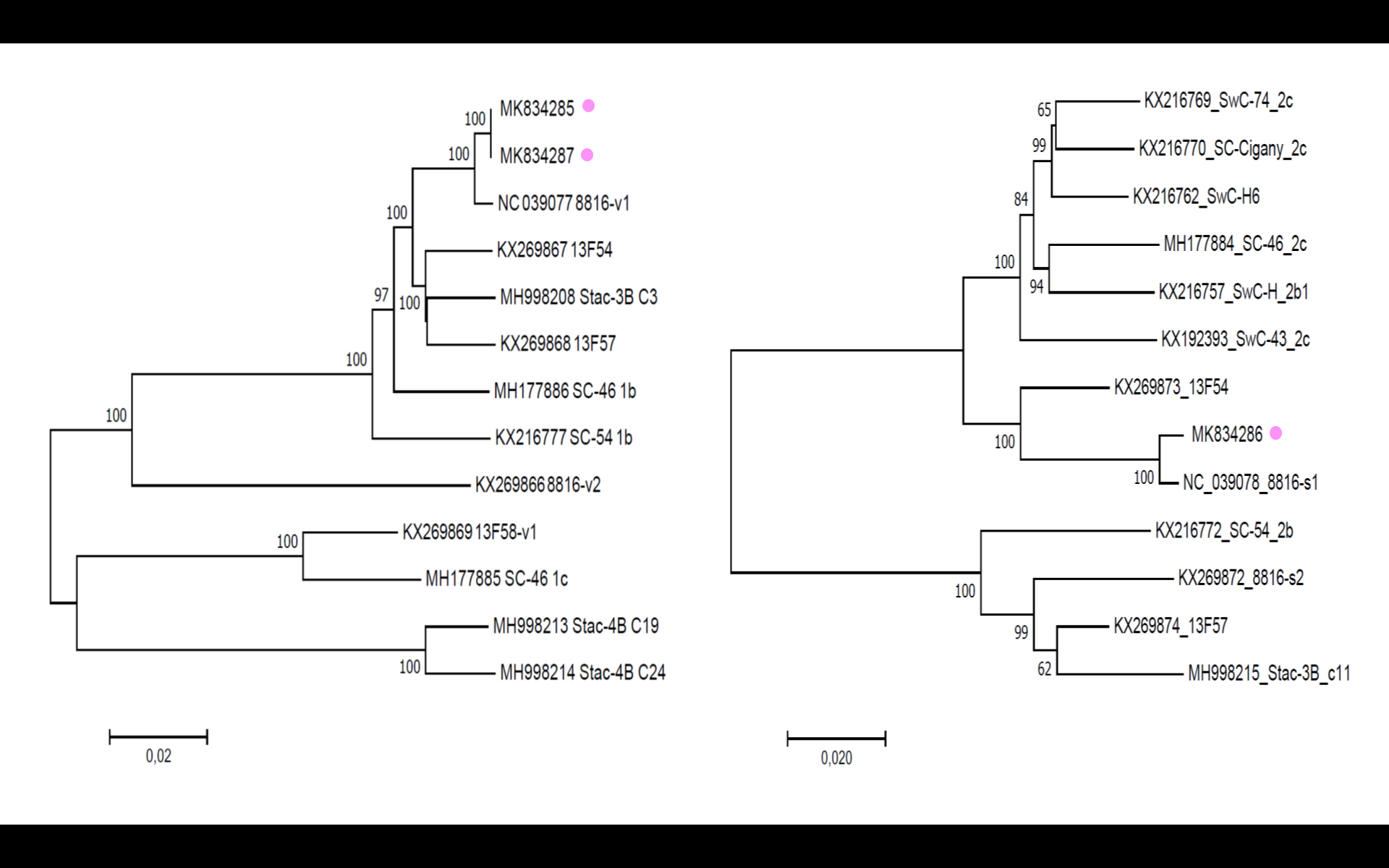


**Figure S4.** Maximum likelihood phylogenetic trees inferred from the full genome RNA1 (left) and RNA2 (right) nucleotide sequences of PrVF isolates (Belgian isolates are indicated with a purple dot). All isolates from Genbank are reported with their accession numbers followed by their names. The numbers above or below each branch are the nonparametric bootstrap (NPB) values given as percentages of 1000 replicates.


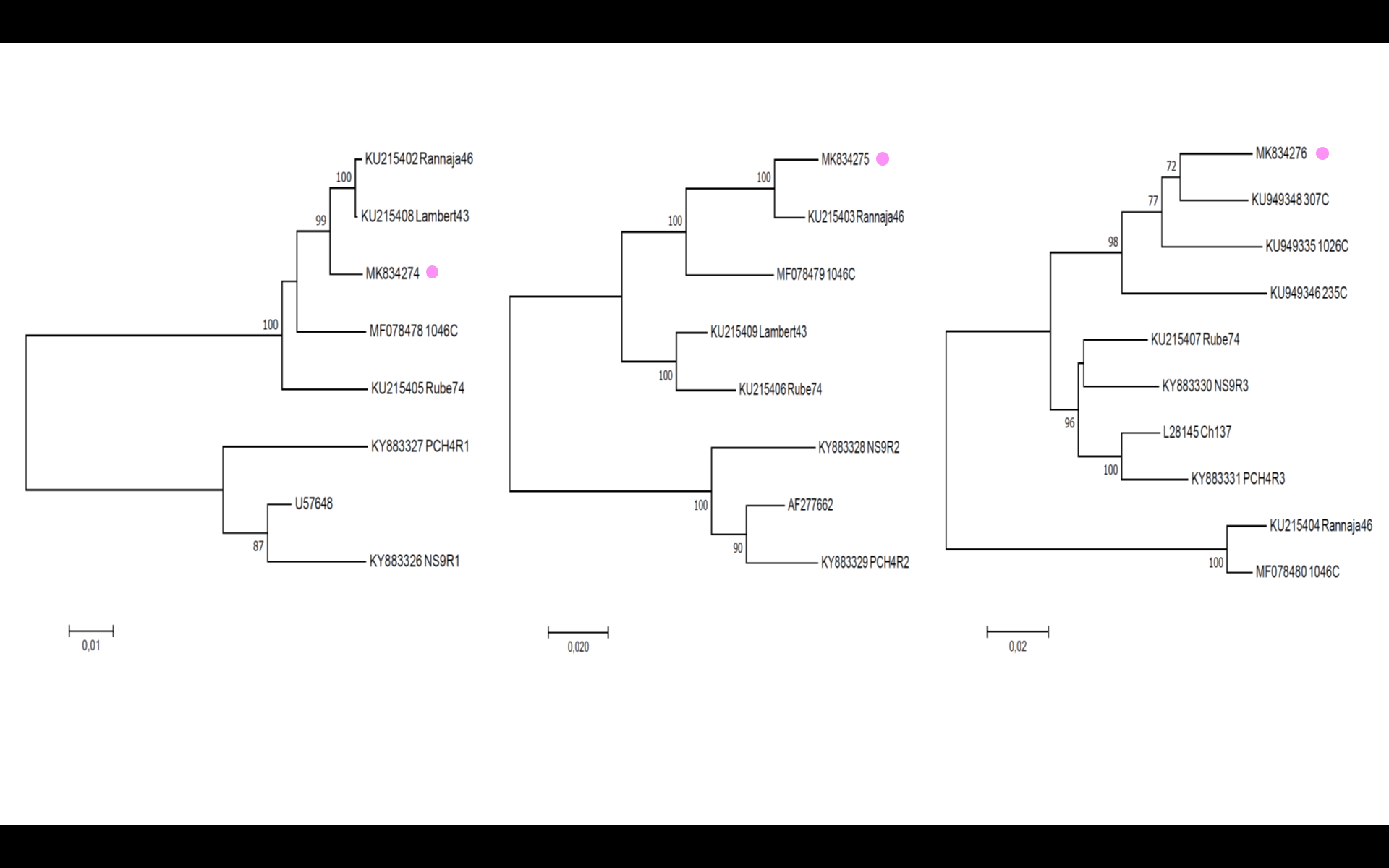


**Figure S5.** Maximum likelihood phylogenetic trees inferred from the full genome nucleotide sequences with RNA1 (left), RNA2 (middle) and RNA3 (right) of PDV isolates (Belgian isolates are indicated with a purple dot). All isolates from Genbank are reported with their accession numbers followed by their names. The numbers above or below each branch are the nonparametric bootstrap (NPB) values given as percentages of 1000 replicates.
